# Navigating the Boundaries of Health and Identity: Medical Students' Perspectives

**DOI:** 10.1111/tct.70028

**Published:** 2025-01-19

**Authors:** S. Bull, S. Danso‐Bamfo, J. Ogden, S. Kumar

**Affiliations:** ^1^ Imperial College London London UK; ^2^ University of Surrey Guildford UK; ^3^ University of Leeds Leeds UK

**Keywords:** health beliefs, medical students, notions of health, self‐care, wellbeing

## Abstract

**Background:**

Medical professionals do not always manage their own health in an optimal way. Research conducted on medical students' response to illness is limited; however, some studies suggest they choose to self‐manage and seek advice outside of the traditional pathways of care. This study aims to understand how medical students perceive and manage their health as they transition through medical school.

**Methods:**

Individual semistructured interviews with 10 medical students from one institution in England were conducted. Interviews explored students' experiences of managing their own physical and mental health and the health of family and friends, and how they could be better supported at medical school. Thematic analysis was conducted.

**Results:**

Four themes were described reflecting: the different sources of learning that students used to construct their notion of health and how these changed during their training; how these notions of health influenced the practices that students used for good and bad self‐care; how they held multiple identities of a lay person and somebody with medical status that could result in conflicts and tensions; finally, students suggested supportive practices including creating a safe space for reflection and the discussion of conflict.

**Discussion:**

Becoming a doctor can result in many tensions as students develop increasingly medicalised notions of health that may lead to poor self‐care. Creating spaces within the curriculum to discuss the privileges and tensions that arise from training to be a doctor may yield future benefits for the medical profession and patient care.

## 1 Introduction

The literature is replete with evidence suggesting that members of the medical profession do not always manage their own health and wellbeing in an optimal way [1, 2, 3, 4, 5]. There are significant rates of self‐referral, self‐prescribing, and informal consultations with colleagues rather than with their health practitioner [2, 3, 6], with some doctors feeling that it is acceptable practice to treat chronic health conditions themselves [7]. In recognition of the potential and prevalence of self‐treatment, regulators of the profession have stipulated codes of conduct when it comes to prescribing for oneself and close contacts [8, 9, 10]. Doctors have also been shown to work through periods of sickness and delay seeking help [4, 5, 7, 11] and they are often reluctant to adopt the ‘patient role’ [12]. Furthermore, there is significant concern around the high levels of mental ill‐health in doctors [13, 14], which is being compounded by workforce pressures. For example, in the United Kingdom, nearly all General Practitioners (GPs) are working beyond their expected hours and more than half report that they are exhausted [13]. Workforce pressures with high rates of stress, burn out and mental illness, together with a pervasive culture of self‐treatment and delays in seeking help, provides the conditions for a perfect storm for both our medical workforce, and the care that they can provide to patients [4, 15, 16].

This leads us to consider, where does this culture of poor self‐care (i.e., not maintaining one's health appropriately), originate from and at what stage in their career do clinicians begin to develop these attitudes and maladaptive behaviours of managing and seeking care for their own health? Research shows that medical students, especially those in their clinical years, also choose to seek advice outside of the traditional pathways of care [1, 17, 18, 19]. Students have reported feelings of embarrassment, panic, anxiety around perceptions of triviality, and fears of lack of confidentiality and stigma associated with formal medical treatment [2, 3, 20]. Studies also show that many students experience mental ill‐health whilst at medical school [21] and that their quality‐of‐life (i.e., their physical and social functioning) declines as they progress through their medical degree [22] This may be associated with the intensity of their course [23]. However, medical students also gain specialised knowledge that is immediately applicable to themselves, and whilst evidence is conflicting, a discourse that started in the late 1960s, suggested that medical students may exhibit greater levels of health anxiety than students studying other disciplines [24, 25, 26], coining the phrase ‘medical student syndrome’.

Understanding the aetiology of these early signs of maladaptive response to illness may give us some insights into how to support medical students (our future clinicians) in managing their own health in a more productive way. Whilst there are some existing studies about medical students' beliefs about their health, the exploration of the drivers that underpin this is limited. By adopting a qualitative approach, we aimed to gather in‐depth information about medical student's experiences of (1) how they perceive and manage their own health as they transition through medical school, and what factors influence this? (2) what role they play in providing health advice to members of their family and social networks? and (3) how could they be best supported to manage their health and wellbeing in medical school?

We hope the insights from this study will shape the development of medical school curricula to promote more adaptive responses to illness and a culture of self‐care for medical students that they can take forward into their future professional life.

## 2 Methods

### 2.1 Study Design

We adopted an interpretive approach and a qualitative study design. Data were collected using individual semi‐structured interviews and analysed by inductive thematic analysis [27].

### 2.2 Study Context

The research was conducted in one English university between October 2021 and January 2022. Student participants were in their final 3 years of their medical degree. Demographic information was not collected, as we wanted to enable participants to speak from multiple perspectives and identities, rather than that generated from a simplistic categorisation.

### 2.3 Reflexivity

The research team recognised that they would bring their own perspectives to the research but took care to think about this in relation to the study aims. SB is a non‐clinical medical educator. She is aware that clinical colleagues access healthcare differently to herself and perceives this as having advantages and disadvantages. SDB is a recently qualified General Practitioner (GP). Her parents were clinicians, which she recognises influenced how she accessed healthcare in her formative years. She has also practised medicine outside of the United Kingdom, priming her to the influence of societal attitudes to health and healthcare. SK has worked as a GP over two decades, she has experienced first‐hand, the challenge of supporting friends and family who ask for informal health advice, being expected to offer professional knowledge whilst also being a friend/family member. JO is a specialist in the psychology of health, she was aware that her disciplinary knowledge may shape her interpretation of the participants' narratives.

### 2.4 Method

Students were approached to participate in the study through communication in a student newsletter and through advertisement at teaching sessions. The first 10 students to express an interest in the study were interviewed. Interviews were conducted online and explored participants' views on their health and their approach to managing their health, as well as the health of their family and friends. They were also asked about the ways in which medical schools could provide better support for them (topic guide provided in Table 1). Two clinical educators who were working within the institution conducted the interviews (KE and RP cited in the acknowledgments section). Participants were reassured about confidentiality and encouraged to speak freely. Interviews were audio‐recorded with permission and transcribed verbatim by a professional transcription company.

**TABLE 1:** Topic guide to enquire how medical student participants consider, and manage, their health at medical school?

Topic	Prompt questions
How do medical students perceive health?	How do you define health? What has informed how you define health? Is there anything from your personal background that has shaped how you think about health?
How do medical students manage their health?	Prior to medical school, what support and resources did you use to help manage your health? Since becoming a medical student, do you draw upon the same support and resources? Have you noticed any differences between medical and non‐medical peers in how they manage their health? If you are consulting with a healthcare professional about your health, do you tell them that you are a medical student (why/why not)?
How do medical students support others with health enquiries?	Do your family and friends ever ask you for healthcare advice? If so, how do you approach this, and how does it make you feel?
What support could the medical school provide to students?	What could the medical school do to support medical students to manage their health? Is there anything additional that you would like to tell us about how you manage your health at medical school?

Thematic analysis was conducted through the steps of data familiarisation, coding, and creation of themes [27]. The analysis was inductive. Two researchers (SB and SDB) immersed themselves in the data, reading the transcripts and noting initial impressions (they did not check the accuracy of the transcription against the audio‐recordings of the interviews). They then each independently coded each transcript making electronic notes that identified the unique aspects of participants' experiences. They then collaborated to review the codes and create themes that captured insights from the data. Once the initial thematic analysis was complete, it was discussed by SB, SDB, SK, and JO resulting in further expansion and collapsing of themes and refinement of the descriptors, until consensus was achieved.

### 2.5 Ethical Considerations

Approval was granted from the participating university August 2021 (name withheld to protect anonymity). Participants gave written informed consent.

## 3 Results

**FIGURE 1:**
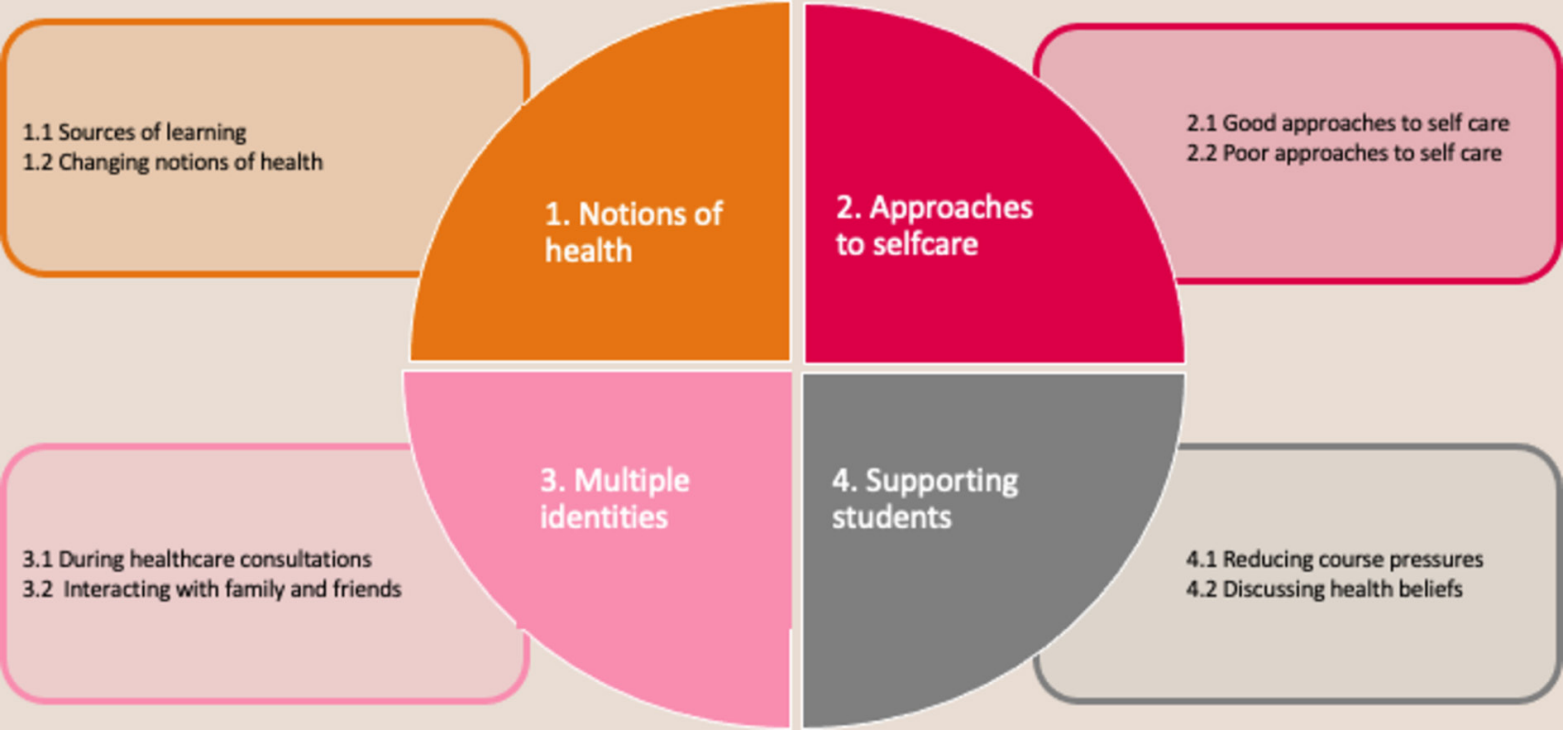
Themes and Subthemes developed from the interviews.

Ten students were interviewed (median interview length 30 min, range 18–39 min). Thematic analysis led to the identification of four themes: (1) notions of health; (2) approaches to self‐care; (3) multiple identities; (4) supportive practices. Each theme had subthemes which are shown (Figure 1) and described below.

Four themes: (1) notions of health; (2) approaches to self‐care; (3) multiple identities; (4) supportive practices.

### 3.1 Theme 1. Notions of Health

Students gained their knowledge, conceptions and beliefs about health from a variety of sources. These were noted to change during student's time at medical school.

Students learnt from a wide range of sources routed initially from their families and communities.

my family is classic South‐Asian, do not bother the doctor P9

Drawing on advice from their family became less frequent as students progressed through medical school, either because students were living more independently of their families, or because their health knowledge surpassed that of family members.

I would not consult family members [now I'm a medical student] just because I feel like I would probably know better … P8

Students used online resources to guide them to make health decisions, but their preference changed from using resources designed for patients, to those intended for healthcare professionals.

I can go to the right resources to read about it. I know where to go on the NHS website, sometimes I'll even look at the NICE guidelines P2

Another prominent source of learning was student's experiences in clinical practice. Understanding the rationale behind clinical decisions and developing an understanding of how the healthcare system works was particularly influential.

Now, I understand the importance of just waiting and monitoring and the rationale behind It. P10

Many students also reported that they asked clinical teachers medical questions that concerned their own health, by inferring that they were asking the question about somebody else or simply out of interest.

you can also ask what it seems like is a random question to a random consultant about something that you are worried you have … I access healthcare like that P3

Students also sought medical opinions from their peers, either for advice, reassurance, or for intellectual interest.

I can go to … [friends who are studying medicine], and they will confirm my suspicions …. and a part of that is just that medical students seem to find it more interesting P4

In terms of changing attitudes to health, students described how before starting medical school their definition of health centred around absence of disease, whereas now it had expanded to being about physical, mental, and social wellbeing.

[health means that] life is enjoyable and meaningful without unnecessary inhibition or unnecessary detriments or barriers to achieving that through physical health, mental health, or social well‐being. P8

### 3.2 Theme 2. Approaches to Self‐Care

Students described the approaches that they took to manage their own health. Many students described that since studying medicine they felt more confident to make health‐decisions, choosing not to seek medical care for diseases that were perceived to be self‐resolving and seeking care when there were known treatment benefits.

Knowledge of disease processes … and what you should be doing and what you should not be doing definitely does play a part P4

They felt more confident to make health‐decisions.

However, medical students also described beliefs and behaviours that would lead to poor self‐care. For example, students expressed the notion that they should ‘be healthy’.

As a medical student you are supposed to be completely fit. I'm not sure exactly what it is, but I do feel like there is a bit of stigma about being unwell. I just feel that I should not be going to the GP very often. P6

Many also trivialised their own ill‐health, by downplaying their illnesses as not being serious enough to require professional care.

Downplaying their illnesses as not being serious enough to require professional care.

I guess I do feel like I should be able to get it sorted myself, but at the back of my mind, I also know that it's probably a better idea to go see a doctor who is actually able to do something for me P7

Frequently they compared their own health issues to those of patients that they had met on placement.

I feel like my problems, compared to what I usually see are very small, so I feel like it might be a bit of a waste of time to go to a GP when it's not life‐threatening or anything like that P6

Other students, however, became hyperaware/anxious about their health, attributing this to their increasing knowledge about the severity of some diseases.

I think that it is because our knowledge base expands so quickly and we know so much about big conditions that are going to kill people … we are more likely to jump to the worst‐case scenario, because we do not have the proper clinical reasoning and the insights which come with experience P8

Many students were cognisant of the importance of health promoting activities yet found it difficult to enact this because of the demands of their course, for example, structured timetable/high workload, a culture of competitiveness, and encountering emotionally charged experiences.

Because we are all ranked against each other and things like that … any hour not spent working is a lost hour … so, then health is very overlooked, because you just do not have time to take care of yourself P2

### 3.3 Theme 3. Multiple Identities

Students described holding identities of both a lay person, and a person with medical knowledge both in relation to being a patient themselves, and when interacting with family and friends about their health.

Some students saw advantages of disclosing that they were a medical student if they were receiving medical care in consultations as this could make the consultations more efficient, and provide them with greater leverage for getting their agenda served.

[the clinician] was like, oh, you are a medical student, you know you have an ear infection, here's some antibiotics … if I was a lay person, it might have been a bit more delayed in terms of when they gave me the antibiotics. P1

Some students saw disadvantages though, as it reduced the explanations that they received:

I often try and hide it, just because if I do want that liberty of just being a patient, and just turning up with a problem and not having it put on me [to explain what I think and what I want]. I still want that luxury of just being cared for … P8

Other students feared being judged as somebody that overthinks things:

I feel if they know immediately that you are a medical student, [that] they sometimes take everything you say with a pinch of salt. Because they might think that … you are putting one and two together and getting six or something P5

There were also many conflicting emotions associated with holding multiple‐identities and being asked to ‘play doctor’, with family and friends. Most students wanted to be supportive yet felt that they may be perceived as dispassionate if they used an approach aligned to that of a healthcare practitioner.

A friend did tell me that [her Father] was having his prostate biopsied. And in my mind, it's like, ‘okay, so we do not know what the outcome is, it might not be cancerous' … But she was slightly offended that I did not react in a … ‘oh no, I'm really sorry’ way. P4

### 3.4 Theme 4. Supportive Practices

This theme described the practices that may support student to manage their health whilst at medical school. The first recommendation was to reduce the pressures experienced during their course. Workload pressures also featured in Theme 2 as they were described as limiting a student's opportunity to engage with health promoting activities. The second recommendation was to provide reflective opportunities for students to discuss how their health behaviours are shaped and change during medical school:

I do not think most medical students actually talk about how they access medical care P6

To provide reflective opportunities for students to discuss how their health behaviours are shaped and change during medical school.

## 4 Discussion

This study provided insights into how medical students perceived and managed their own health and that of others as they transitioned through medical school. It has highlighted how their notions of health changed as they were exposed to different sources of learning; how this can lead to both adaptive and maladaptive self‐care practices; how they experienced tensions through multiple identities as both a lay person and a medical expert; and how they felt the need for additional support to help them navigate the transitions and tensions that occur as they progress through medical school.

Notions of health changed as they were exposed to different sources of learning.

In terms of *notions of health*, participants described how exposure to illness, clinical practice and medical education shifted their beliefs towards a more medicalised model of health in terms of both the causes and treatments of disease. This is reflected in Leventhal's self‐regulatory model [28] and illustrates the dynamic nature of illness beliefs. For example, as medical students receive training in making accurate diagnoses and appropriate management plans, this insider knowledge enabled them to manage their own health in adaptive ways that may well be different to a lay person, for example, following approaches specified in clinical guidelines. At times, however, this insider knowledge came with costs and students described maladaptive responses to illnesses: occasionally becoming overanxious about their health, a concept that has been noticed in other studies and described as ‘medical student syndrome’ [24, 25, 26] or at the other extreme trivialising the importance of seeking healthcare and perceiving a stigma associated with seeking treatment and adopting a patient role [2, 3, 12, 20]. Such behaviour is not unique to medical students, nursing students also request physician consultations less frequently during their training [29]. Further, counsellors have also been shown to struggle with self‐care [30], which has led to a call from researchers to provide a more compassionate culture during training of future health professionals, which encourages self‐care throughout their careers [31].

Students also described the challenges posed by holding multiple identities which led to transitional shift in identity from lay person, who was also a relative or friend, to that of medical expert as students transitioned through medical school. This emerging medical status created a tension as they navigated managing their own health and that of others. Such tension reflects the need for appropriate regulation [8, 9, 10] that provides the boundaries to support medical students and qualified health professionals to behave in a professional way both to themselves and those around them.

Finally, many students recommended ways to facilitate their transition through medical school. In particular, students suggested creating reflective curricular spaces to discuss the privileges and tensions that arise as a medical student managing their own health. Such interventions could be implemented at the start of medical school but be ramped up shortly after students have entered their clinical years, as this was considered the period of greatest transition.

## 5 Conclusion

This study highlights how medical students changed their notions of heath and health management practices as they transitioned through medical school. This was often underpinned by tensions around identity and the shift from lay person to medical expert that may result in increasingly maladaptive approaches to self‐care. At present, such tensions are neglected by the medical curriculum and students feel unsupported. Given the potential impact of these behaviours on medical students and clinician's own health, and their ability to care for others appropriately, curricula need to include supportive reflective spaces for students to openly discuss their self‐care practices and the inevitable tensions and conflicts they experience during their training.

## 6 Strengths and Weakness

The semistructured nature of the interviews provided a forum for students to highlight concerns and behaviours that were important to them. The prompts were developed following pilot interviews with medical students and the clinical members of the research team reflecting on their own experiences. Resource constraints meant that only 10 students were interviewed, and these were all enrolled in one UK medical school. Some of findings therefore may be bounded to the context of that institution.

## 7 Further Work

This study highlights some of the tensions encountered as medical students progress through their training and illustrates the potential impact of these tensions on both their own self‐care and the care of others. Further studies are needed to elucidate the root causes of why medical students do not access health care when needed, nor feel able to prioritise health promoting activities. Whilst students mentioned that reducing pressures encountered on their course would be helpful, we must be cognisant of realistically preparing future doctors to manage their own heath, even when workloads are high.

Realistically preparing future doctors to manage their own heath, even when workloads are high.

## Disclosure

The views expressed in this publication are those of the authors and not necessarily those of the NHS, the NIHR or the Department of Health.

## Data Availability

The data that support the findings of this study are available on request from the corresponding author. The data are not publicly available due to privacy or ethical restrictions.
